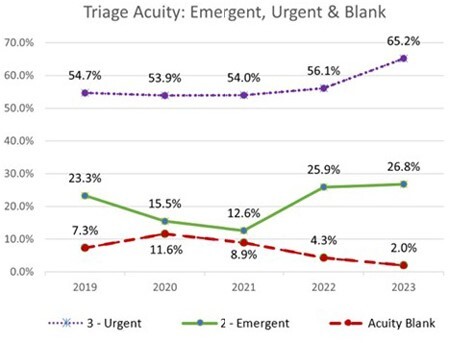# 24 Bleeding Money: Improving Triage and Documentation for Burn Patients in the Emergency Department

**DOI:** 10.1093/jbcr/irae036.024

**Published:** 2024-04-17

**Authors:** Stacey Richerbach, Tiffany Hockenberry, Kevin N Foster

**Affiliations:** Arizona Burn Center Valleywise Health, Phoenix, AZ; Arizona Burn Center Valleywise Health, Phoenix, AZ; Arizona Burn Center Valleywise Health, Phoenix, AZ

## Abstract

**Introduction:**

Triage acuity is a critical factor in determining resource allocation and prioritizing patient care. Additionally, documented triage acuities are correlated with professional charges and billing codes. Within the emergency department (ED), burn patients comprise a unique population. For a patient with a burn the insult is apparent, however, the resultant physiologic response may be overlooked by ED clinicians. For example, patients with burns may present with severe pain or elevated heart rate, yielding a high acuity level. In 2020, our Burn Center observed an increase in undocumented triage acuity for burn patients to 11.6%. As a result, we instituted a multidisciplinary quality improvement (QI) approach to improve triage related documentation and charge capture, and prioritization of care for patients with burns. The purposes of this project were to pinpoint education needs of nursing staff and improve documentation of triage acuity per the Emergency Severity Index (ESI).

**Methods:**

A survey was disseminated to Burn ED nursing staff to identify barriers to triage documentation. QI interventions included a collaboration between Nursing, Informatics, and Quality Assurance to overcome barriers related to staff knowledge, workflow of the electronic health record (EHR), and dissemination of performance feedback. Education was provided via targeted training, pre-shift nursing huddle, weekly newsletters, and educational posters. Modifications were made to the EHR software modify the order of items in Triage list to avoid bypass of acuity documentation and to embed both a visual algorithm and text descriptors for ESI acuity determination. Quality Assurance furnished reports monthly, enabling nursing leadership to expedite feedback and identify needs for staff-specific education. Triage acuity metrics were added to the quality board. Performance was analyzed from January 2019 through June 2023, and was grouped annually.

**Results:**

Following the QI project, undocumented triage acuity was reduced by 9.6%. Further, we noted an increase in both emergent (level 2) and urgent (level 3) acuity assignments by 11.3% for each.

**Conclusions:**

The accurate assignment of triage acuity using scoring delineated by the ESI impacts both care and compensation. Our multidisciplinary quality improvement project was successful, reducing undocumented triage acuity by 9.7%. Further, we discovered a need to reinforce education related to high ESI triage acuity qualifiers, such as extreme pain and elevated heart rate. In doing so, we observed an increase in documentation of higher acuity levels for patients with burns (emergent and urgent) by 11.2% and 11.3%.

**Applicability of Research to Practice:**

Further research is needed to quantify the impact of triage acuity assignment on charge capture.